# Pioglitazone Improves the Function of Human Mesenchymal Stem Cells in Chronic Kidney Disease Patients

**DOI:** 10.3390/ijms20092314

**Published:** 2019-05-10

**Authors:** Yeo Min Yoon, Jun Hee Lee, Chul Won Yun, Sang Hun Lee

**Affiliations:** 1 Medical Science Research Institute, Soonchunhyang University Seoul Hospital, Seoul 336-745, Korea; yoonboo15@naver.com (Y.M.Y.); j-school@hanmail.net (J.H.L.); skydbs113@naver.com (C.W.Y.); 2 Department of Biochemistry, Soonchunhyang University College of Medicine, Cheonan 330-930, Korea

**Keywords:** cellular prion protein, chronic kidney disease, endoplasmic reticulum stress, mesenchymal stem cells, mitochondria, proliferator-activated receptor gamma coactivator 1-alpha

## Abstract

Mesenchymal stem cells (MSCs) are optimal sources of autologous stem cells for cell-based therapy in chronic kidney disease (CKD). However, CKD-associated pathophysiological conditions, such as endoplasmic reticulum (ER) stress and oxidative stress, decrease MSC function. In this work, we study the protective effect of pioglitazone on MSCs isolated from CKD patients (CKD-MSCs) against CKD-induced ER stress. In CKD-MSCs, ER stress is found to induce mitochondrial reactive oxygen species generation and mitochondrial dysfunction. Treatment with pioglitazone reduces the expression of ER stress markers and mitochondrial fusion proteins. Pioglitazone increases the expression of cellular prion protein (PrP^C^) in CKD-MSCs, which is dependent on the expression levels of proliferator-activated receptor gamma coactivator 1-alpha (PGC-1α). Treatment with pioglitazone is found to protect CKD-MSCs against reactive oxygen species generation, aberrant mitochondrial oxidative phosphorylation of complexes I and IV, and aberrant proliferation capacity through the PGC-1α-PrP^C^ axis. These results indicate that pioglitazone protects the mitochondria of MSCs from CKD-induced ER stress. Pioglitazone treatment of CKD-MSCs may be a potential therapeutic strategy for CKD patients.

## 1. Introduction

Chronic kidney disease (CKD) is a global health concern due to the increasing prevalence (8–16%) of the disease [1]. CKD is characterized by the gradual loss of function over a period of months to years. By contrast, acute kidney injury (AKI) is defined as a kidney disease with rapid loss of renal function. Both CKD and AKI are considered risk factors for the development of diseases that progress from AKI to CKD or CKD to AKI [2,3]. CKD is associated with several pathophysiological conditions, including type 1 or type 2 diabetes, high blood pressure, glomerulonephritis, interstitial nephritis, vesicoureteral reflux, and kidney infection. These conditions can impair kidney function for long periods of time [1,4,5]. Progression of CKD is associated with several complications, such as swelling of the arms and legs, cardiovascular disease, weak bones, and a damaged central nervous system [5,6,7]. In CKD patients, production of reactive oxygen species (ROS) is increased by uremic toxins [8,9,10], leading to the generation of abnormal biomolecules, which damage cells and affect tissue function [11]. Uremic toxins also induce the activation of endoplasmic reticulum (ER) stress [9,12,13]. These pathophysiological conditions are major hurdles for the clinical application of stem cell- and progenitor cell-based therapies.

Mesenchymal stem cells (MSCs) are a promising source for autologous stem cells in the treatment of CKD. Bone marrow-derived MSCs (BM-MSCs) differ in the expression of some surface markers and phenotype compared with adipose-obtained MSCs (AD-MSCs) [14,15]. However, these differences can be slight, and similarities between BM-MSCs and AD-MSCs include self-renewal, multipotency, multi-lineage differentiation, and secretion of cytokines [16,17,18,19,20]. In addition, a previous study reported that MSCs obtained from 10 patients with end-stage renal disease (ERSD) displayed no difference in their capacity of differentiation compared to healthy-MSCs [21]. The collective results supported the clinical potential of MSCs derived from patients.

Although autologous cell therapy is an essential therapeutic strategy for CKD patients, the therapeutic potential of AD-MSCs and BM-MSCs isolated from CKD patients (CKD-MSCs) is reduced due to the impairment of cell functions by uremic toxins [8,10,22,23]. CKD-induced ER stress and production of ROS results in impaired mitochondrial function in MSCs of CKD patients [20,24]. Since these CKD-induced pathophysiological conditions suppress the therapeutic potential of autologous MSCs in CKD patients, a novel strategy for the development of functional autologous MSCs is urgently needed.

Normal cellular prion protein (PrP^C^) is a glycoprotein which enhances cell adhesion with components of the extracellular matrix, such as laminin and vitronectin [25,26]. PrP^C^ is associated with proliferation, survival, and regulation of signaling pathways [27,28]. We have previously demonstrated that PrP^C^ plays a central role in protection against oxidative stress in an ischemia injury model via regulation of anti-oxidant enzymes and reduction of ER stress [13]. Pioglitazone is a thiazolidinedione drug that is prescribed for the treatment of type 2 diabetes. The drug also enhances stem cell function by protecting cells against apoptosis, improving cell differentiation, and promoting cell proliferation by enhancing mitochondrial biogenesis. However, whether pioglitazone protects CKD-MSCs against ER stress is unknown.

In this study, we investigate the role of pioglitazone in protecting the mitochondrial function and proliferation capacity of CKD-MSCs against CKD-induced ER stress through regulation of PrP^C^ expression.

## 2. Results

### 2.1. Chronic Kidney Disease (CKD) Induces Endoplasmic Reticulum (ER) Stress and Mitochondrial Dysfunction in Human Mesenchymal Stem Cells (MSCs)

**Figure 1:**
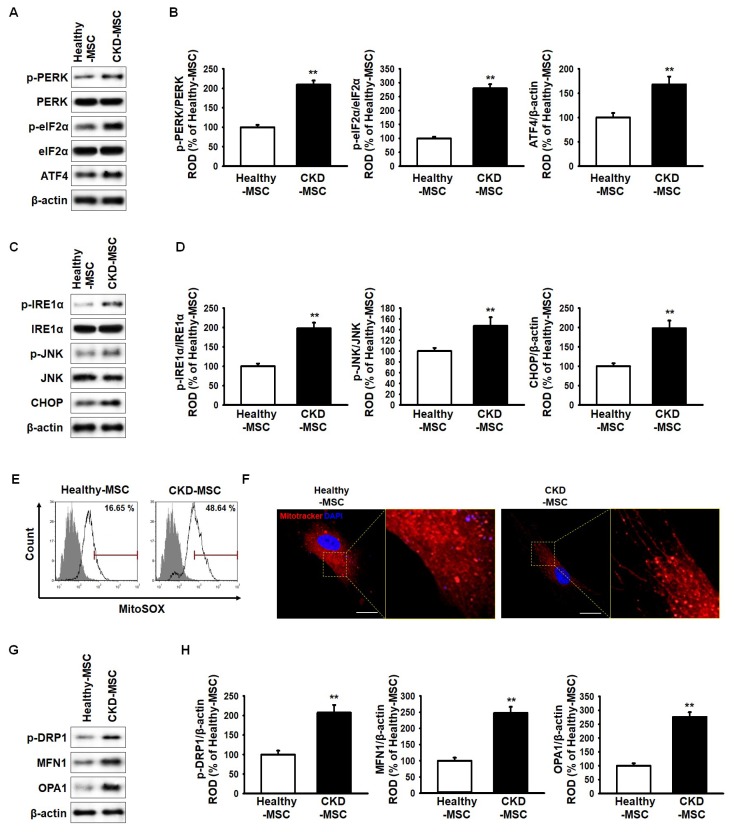
Effect of chronic kidney disease (CKD)-induced endoplasmic reticulum (ER) stress on mitochondria in mesenchymal stem cells (MSCs) isolated from CKD patients. (**A**) Expression of phospho-protein kinase R-like endoplasmic reticulum kinase (p-PERK), protein kinase R-like endoplasmic reticulum kinase (PERK), phospho-eukaryotic translation initiation factor 2 alpha (p-eIF2α), eukaryotic translation initiation factor 2 alpha (eIF2α), and activating transcription factor 4 (ATF4) in human healthy-MSCs and MSCs isolated from CKD patients (CKD-MSCs); (**B**) The expression levels of p-PERK, p-eIF2α, and ATF4 were determined relative to the expression levels of PERK, eIF2α, and β-actin, respectively; (**C**) Expression of phospho-inositol-requiring enzyme 1 alpha (p-IRE1α), inositol-requiring enzyme 1 alpha (IRE1α), phospho-c-Jun N-terminal kinase (p-JNK), c-Jun N-terminal kinase (JNK), and CCAAT-enhancer-binding proteins (C/EBP) homologous protein (CHOP) in healthy-MSCs and CKD-MSCs; (**D**) The expression levels of p-IRE1α, p-JNK, and CHOP were determined relative to the expression levels of IRE1α, JNK, and β-actin, respectively; (**E**) Mitochondria superoxide (MitoSOX)-positive MSCs isolated from healthy individuals (healthy-MSCs) and CKD-MSCs were quantified by fluorescence-activated cell sorting analysis; (**F**) Mitochondrial morphology was analyzed by immunofluorescence staining using MitoTracker (red). The scale bar = 100 μm; (**G**) Expression of phospho-dynamin 1-like protein (p-DRP1), mitofusin-1 (MFN1), and dynamin-like 120 kDa protein (OPA1) in healthy-MSCs and CKD-MSCs; (**H**) The expression levels were determined relative to the expression levels of β-actin. Values represent the mean ± standard error of the mean (SEM). ** *p* < 0.01 versus healthy-MSCs.

CKD-induced endoplasmic reticulum (ER) stress in human MSCs was evaluated by measuring the expression levels of ER stress markers, protein kinase R-like endoplasmic reticulum kinase (PERK), eukaryotic translation initiation factor 2 alpha (eIF2a), activating transcription factor 4 (ATF4), inositol-requiring enzyme 1 alpha (IRE1a), c-Jun N-terminal kinase (JNK), and CCAAT-enhancer-binding proteins (C/EBP) homologous protein (CHOP). As shown in Figure 1A–D, expression of these ER stress markers in CKD-MSCs was significantly higher when compared to their expression in healthy-MSCs. A previous study reported that ROS generation is increased in CKD patients by uremic toxin [8]. Mitochondria superoxide (MitoSOX)-based flow cytometry analysis staining was performed to investigate the generation of mitochondrial ROS in healthy-MSCs and CKD-MSCs. Mitochondrial ROS production in CKD-MSCs was higher than that observed in healthy-MSCs (Figure 1E). To further assess the mitochondrial morphology in healthy-MSCs and CKD-MSCs, MitoTracker immunofluorescence staining was performed, revealing increased mitochondrial fusion in CKD-MSCs (Figure 1F). In addition, the expressions of mitochondrial fusion markers, phospho-dynamin 1-like protein (p-DRP1), mitofusin-1 (MFN1), and dynamin-like 120 kDa protein (OPA1) were significantly higher in CKD-MSCs compared to their expressions in healthy-MSCs (Figure 1G,H). These results indicate that CKD induced ER stress in the human MSCs, resulting in mitochondrial dysfunction.

### 2.2. Pioglitazone Prevents Generation of Reactive Oxygen Species (ROS) via the Proliferator-Activated Receptor Gamma Coactivator 1-Alpha Cellular Prion Protein (PGC-1α-PrP^C^) Axis

**Figure 2:**
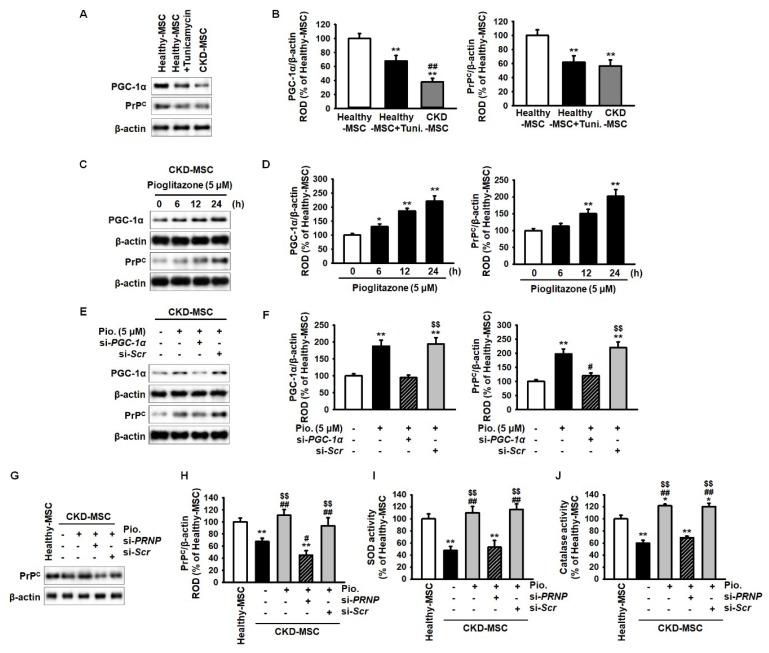
Pioglitazone increases anti-oxidant activity through the proliferator-activated receptor gamma coactivator 1-alpha cellular prion protein (PGC-1α-PrP^C^) axis. (**A**) Expression of proliferator-activated receptor gamma coactivator 1-alpha (PGC-1α) and cellular prion protein (PrP^C^) in MSCs isolated from healthy individuals (healthy-MSCs), tunicamycin-treated healthy-MSCs (5 μg/mL for 4 h), and CKD-MSCs; (**B**) The expression levels were determined relative to expression levels of β-actin. Values represent the mean ± SEM. ** *p* < 0.01 versus healthy-MSC, ## *p* < 0.01 versus tunicamycin-treated healthy-MSCs (healthy-MSC + Tuni.); (**C**) Expression of PGC-1α and PrP^C^ in pioglitazone-treated CKD-MSCs (5 μM for 24 h); (**D**) The expression levels were determined relative to the expression levels of β-actin. Values represent the mean ± SEM. * *p* < 0.05, ** *p* < 0.01 versus non-treated CKD-MSCs; (**E**) Expression of PGC-1α and PrP^C^ after treatment with pioglitazone (Pio.) and *PGC-1α* short interfering (si)RNA (si-*PGC-1α*); (**F**) The expression levels were determined relative to the expression levels of β-actin. Values represent the mean ± SEM. ** *p* < 0.01 versus non-treated CKD-MSCs, # *p* < 0.05 versus pioglitazone-treated CKD-MSCs, $$ *p* < 0.01 versus pioglitazone-treated CKD-MSCs pretreated with si-*PGC-1α*; (**G**) Expression of PrP^C^ in healthy-MSCs and CKD-MSCs after treatment with pioglitazone and *PRNP* siRNA (si-*PRNP*); (**H**) The expression levels were determined relative to the expression levels of β-actin. Values represent the mean ± SEM. ** *p* < 0.01 versus healthy-MSCs, # *p* < 0.05, ## *p* < 0.01 versus non-treated CKD-MSCs, $$ *p* < 0.01 versus pioglitazone-treated CKD-MSCs pretreated with si-*PRNP*; (**I**,**J**) Analysis of superoxide dismutase (SOD) (**I**) and catalase activity (**J**) in healthy-MSCs and CKD-MSCs after treatment with pioglitazone and si-*PRNP*. Values represent the mean ± SEM. * *p* < 0.05, ** *p* < 0.01 versus healthy-MSCs, ## *p* < 0.01 versus non-treated CKD-MSCs, $$ *p* < 0.01 versus pioglitazone-treated CKD-hMSCs pretreated with si-*PRNP*. Legend: si-*Scr*, scrambled siRNA.

Pioglitazone reduces cellular ROS by increasing the expression of proliferator-activated receptor gamma coactivator 1-alpha (PGC-1α) and enhances mitochondrial biogenesis [29]. In addition, our previous study has indicated that PrP^C^ effectively protects MSCs against oxidative stress [13]. Thus, we assessed the expression of PGC-1α and PrP^C^ in human MSCs under ER stress by evaluating the expression of PGC-1α and PrP^C^ in healthy-MSCs, healthy-MSCs treated with tunicamycin (an inducer of ER stress), and CKD-MSCs. The expression levels of these proteins were significantly lower in healthy-MSCs treated with tunicamycin (5 μg/mL) for 4 h and CKD-MSCs, compared to their expression in healthy-MSCs (Figure 2A,B). These results indicate that ER stress decreased the expression of PGC-1α and PrP^C^. Furthermore, we investigated whether pioglitazone (5 μM for 24 h) regulated the expression of PGC-1α and PrP^C^ in CKD-MSCs. The expression of PGC-1α and PrP^C^ was significantly higher in CKD-MSCs treated with pioglitazone compared to their expression in untreated CKD-MSCs (Figure 2C,D). To further explore the relationship between PGC-1α and PrP^C^, we evaluated the effects of the knockdown of PGC-1α and PrP^C^ in CKD-MSCs. PGC-1α knockdown significantly decreased the expression of PrP^C^ (Figure 2E,F). In addition, knockdown of PRioN Protein (PRNP) significantly reduced the activities of superoxide dismutase (SOD) and catalase (Figure 2G–J). However, treatment with pioglitazone blocked the inhibition of PGC-1α and PrP^C^ expression and preserved the activity of anti-oxidant enzymes during ER stress. These findings suggest that pioglitazone can protect the activity of anti-oxidant enzymes against ER stress by regulating PGC-1α and PrP^C^ expression.

### 2.3. Pioglitazone Inhibits ER Stress and ROS Production through the Expression of Normal Cellular Prion Protein (PrP^C^)

**Figure 3:**
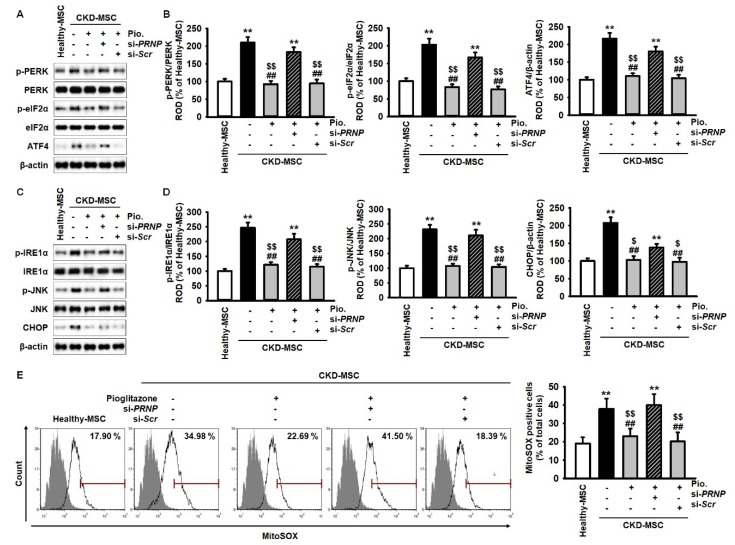
Pioglitazone protects against CKD-induced ER stress and reactive oxygen species (ROS) generation in MSCs isolated from CKD patients through the expression of PrP^C^. (**A**) Expression of p-PERK, PERK, p-eIF2α, eIF2α, and ATF4 in healthy-MSCs and CKD-MSCs after treatment with Pio. and si-*PRNP*; (**B**) The expression levels of p-PERK, p-eIF2α, and ATF4 were determined relative to the expression levels of PERK, eIF2α, and β-actin, respectively; (**C**) Expression of p-IRE1α, IRE1α, p-JNK, JNK, and CHOP in healthy-MSCs and CKD-MSCs after treatment with pioglitazone and si-*PRNP*; (**D**) The expression levels of p-IRE1α, p-JNK, and CHOP were determined relative to the expression levels of IRE1α, JNK, and β-actin, respectively. p-IRE1α, and P-JNK, and expression of ATF4, and CHOP in healthy-MSCs, and treatment CKD-MSCs with or without pioglitazone, and after pretreatment of pioglitazone-treated CKD-MSCs with si-*PRNP*; (**E**) MitoSOX-based flow cytometry analysis in healthy-MSCs and CKD-MSCs after treatment with pioglitazone and si-*PRNP*. Values represent the mean ± SEM. ** *p* < 0.01 versus healthy-MSCs, ## *p* < 0.01 versus non-treated CKD-MSCs, $ *p* < 0.05, $$ *p* < 0.01 versus pioglitazone-treated CKD-MSCs pretreated with si-*PRNP*.

We assessed the expression of ER stress markers in CKD-MSCs after treatment with pioglitazone to evaluate the protective effects of pioglitazone against ER stress via PrP^C^ expression. Expressions of phospho-protein kinase R-like endoplasmic reticulum kinase (p-PERK), p-eIF2α, ATF4, p-IRE1α, phospho-c-Jun N-terminal kinase (p-JNK), and CHOP were significantly higher in CKD-MSCs compared to their expression in healthy-MSCs. Additionally, pioglitazone significantly inhibited the expression of the ER stress markers (Figure 3A–D). However, PRNP knockdown blocked the effects of pioglitazone in CKD-MSCs (Figure 3A–D). MitoSOX-based flow cytometry was performed to establish the effect of pioglitazone on mitochondrial ROS generation in CKD-MSCs. The production of mitochondrial ROS was significantly higher in CKD-MSCs compared to healthy-MSCs (Figure 3E). However, pioglitazone inhibited the production of mitochondrial ROS in CKD-MSCs and PrP^C^ knockdown suppressed the protective effect of pioglitazone against ER stress-induced oxidative stress (Figure 3E). These data indicate that pioglitazone can suppress ER stress-mediated mitochondrial ROS generation in CKD-MSCs by regulating the expression of PrP^C^.

### 2.4. Pioglitazone Enhances Mitochondrial Function in MSCs isolated from CKD patients (CKD-MSCs) by the Upregulation of PrP^C^

**Figure 4:**
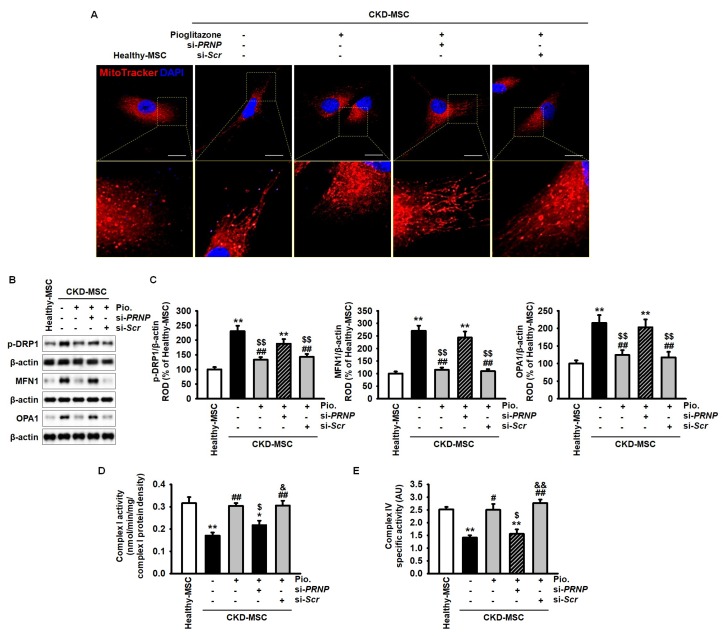
Pioglitazone enhances mitochondrial biogenesis and oxidative phosphorylation via PrP^C^ expression. (**A**) Analysis of mitochondrial morphology by immunofluorescence staining using MitoTracker (red) in MSCs isolated from healthy-MSCs and CKD-MSCs after treatment with Pio. and si-*PRNP*. Scale bar = 100 μm; (**B**) Expression of p-DPR1, MFN1, and OPA1 in healthy-hMSCs and CKD-MSCs after treatment with pioglitazone and si-*PRNP*; (**C**) The expression levels were determined relative to the expression levels of β-actin. Values represent the mean ± SEM. ** *p* < 0.01 versus healthy-MSCs, ## *p* < 0.01 versus non-treated CKD-MSCs, $$ *p* < 0.01 versus pioglitazone-treated CKD-MSCs pretreated with si-*PRNP*; (**D**,**E**) Activity of complex I (**D**) and IV (**E**) in healthy-MSCs and CKD-MSCs after treatment with pioglitazone and si-*PRNP*. Values represent the mean ± SEM. * *p* < 0.05, ** *p* < 0.01 versus healthy-MSCs, # *p* < 0.05, ## *p* < 0.01 versus non-treated CKD-MSCs, $ *p* < 0.05 versus pioglitazone-treated CKD-MSCs, & *p* < 0.05, && *p* < 0.01 versus pioglitazone-treated CKD-MSCs pretreated with si-*PRNP*.

We measured the mitochondrial dynamics of CKD-MSCs using MitoTracker to assess the role of pioglitazone in mitochondrial biogenesis. Pioglitazone protected CKD-MSCs against ER stress-induced mitochondrial fusion by upregulating PrP^C^ expression (Figure 4A). Furthermore, we assessed the expression of the mitochondrial fusion proteins p-DRP1, MFN1, and OPA1 in CKD-MSCs after treatment with pioglitazone. The expression levels of these proteins were significantly higher in CKD-MSCs compared to their expression levels in healthy-MSCs. Treatment with pioglitazone inhibited the increased levels of these proteins in CKD-MSCs by regulating the expression of PrP^C^ (Figure 4B,C). To further verify whether pioglitazone could protect CKD-MSCs against dysfunction of mitochondrial oxidative phosphorylation, we analyzed the activity of mitochondrial complexes I and IV in CKD-MSCs after treatment with pioglitazone. The activity of complexes I and IV was significantly lower in CKD-MSCs when compared to their activity in healthy-MSCs (Figure 4D,E). However, treatment of CKD-MSCs with pioglitazone recovered the activity of complexes I and IV, which was dependent on the expression of PrP^C^ (Figure 4D,E). These results suggest that pioglitazone protects mitochondrial function in CKD-MSCs against ER stress by regulating the expression of PrP^C^.

### 2.5. Pioglitazone Increases Cell Proliferation of CKD-MSCs

**Figure 5:**
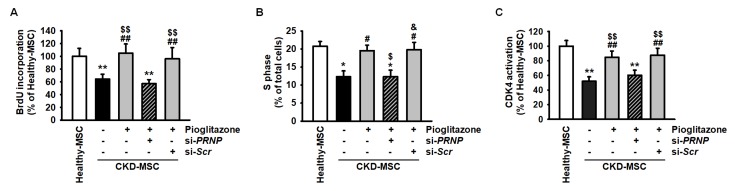
Effect of pioglitazone on the proliferation of CKD-MSCs via the level of PrP^C^. (**A**) 5-bromo-2′-deoxyuridine (BrdU) assay in healthy-MSCs and CKD-MSCs after treatment with Pio. and si-*PRNP*. Values represent the mean ± SEM. ** *p* < 0.01 versus healthy-MSCs, ## *p* < 0.01 versus non-treated CKD-MSCs, $$ *p* < 0.01 versus pioglitazone-treated CKD-MSCs pretreated with si-*PRNP*; (**B**) The percentage of S phase in healthy-MSCs and CKD-MSCs after treatment with pioglitazone and si-*PRNP*. Values represent the mean ± SEM. * *p* < 0.05 versus healthy-MSCs, # *p* < 0.05 versus non-treated CKD-MSCs, $ *p* < 0.05 versus pioglitazone-treated CKD-MSCs, & *p* < 0.05 versus pioglitazone-treated CKD-MSCs pretreated with si-*PRNP*; (**C**) Activity of CDK4 in healthy-MSCs and CKD-MSCs after treatment with pioglitazone and si-*PRNP*. Values represent the mean ± SEM. ** *p* < 0.01 versus healthy-MSCs, ## *p* < 0.01 versus non-treated CKD-MSCs, $$ *p* < 0.01 versus pioglitazone-treated CKD-MSCs pretreated with si-*PRNP*.

The major characteristics of MSC are self-renewal and multipotent differentiation [30]. We assessed the proliferation capacity of CKD-MSCs after treatment with pioglitazone. The proliferation capacity was significantly lower in CKD-MSCs compared to the proliferation capacity of healthy-MSCs (Figure 5A). CKD-MSCs treated with pioglitazone recovered their proliferation capacity to a level observed in healthy-MSCs (Figure 5A). However, PRNP knockdown blocked the effect of pioglitazone on the proliferation capacity of CKD-MSCs (Figure 5A). Additionally, the S phase and CDK4 activation were significantly lower in CKD-MSCs compared to their activation in healthy-MSCs. Pioglitazone protected against ER stress-induced inhibition of proliferation capacity in CKD-MSCs, which was dependent on PrP^C^ (Figure 5B,C). These findings indicate that pioglitazone increases cell proliferation of CKD-MSCs through the PrP^C^-CDK4 signal axis.

## 3. Discussion

Mitochondrial dysfunction is a risk factor for the aggravation of CKD patients via reducing the autologous function of MSCs. Our previous studies have demonstrated that uremic toxicity, similar to that observed in CKD, increases ER stress-induced apoptosis [9,20,31]. In addition, a recent study has shown that several risk factors, which include ER stress, oxidative stress, and dysfunction mitochondria, contribute independently to the development of both AKI and CKD [2]. Another previous study demonstrated that mitochondrial dysfunction associated with ER stress in CKD-MSCs is due to deficient PrP^C^ [31]. Although the characteristics of stem cells differ depending on donor age, gender, body mass index, and therapeutic period, we have previously demonstrated that AD-MSCs derived from CKD patients display decreased expression of PrP^C^ and that CKD patients have low serum levels of PrP^C^ [31,32,33,34]. Treatment of CKD-MSCs with pioglitazone inhibits CKD-induced ER stress and protects against mitochondrial dysfunction by regulating the PGC-1α-PrP^C^ axis.

PGC-1α is involved in the regulation of mitochondrial biogenesis. PGC-1α regulates PrP^C^ expression in pioglitazone-treated CKD-MSCs, resulting in enhanced mitochondrial function. Pioglitazone is a member of the thiazolidinedione class of drugs, which target various metabolic pathways in diabetes [35,36]. The drug alters mitochondrial metabolism by activating PGC-1α signaling for a number of transcription regulatory proteins [37]. PGC-1α is a transcriptional regulator of mitochondrial biogenesis and also regulates mitochondrial respiratory complexes [38]. The reduction in PGC-1α expression results in decreased numbers of mitochondria, attenuated mitochondrial function, and aberrant mitochondrial dynamics [39,40]. CKD patients have also exhibited abnormal mitochondrial morphology [41,42,43]. In a CKD 5/6 nephrectomy mouse model, ROS generation was augmented after exposure to uremic toxin and body weight and skeletal muscle were reduced by the reduced expression of PGC-1α [44]. These findings implicate PGC-1α as a key molecule for mitochondrial function and cell physiology in CKD. Pioglitazone-induced PGC-1α upregulates the expression of PrP^C^. Additionally, we have demonstrated that mitochondrial function and the activity of anti-oxidant enzymes are regulated by PGC-1α-PrP^C^ axis in CKD-MSCs. These data suggest that pioglitazone promotes mitochondrial function and an anti-oxidant effect in MSCs against a CKD-induced pathophysiological condition through PGC-1α-PrP^C^ signal transduction.

Previous studies have demonstrated that PrP^C^ regulates the proliferation and differentiation of neural precursor cells and promotes self-renewal in hematopoietic stem cells [45,46,47]. In this study, pioglitazone-induced upregulation of PrP^C^ protected CKD-MSCs against ER stress and mitochondrial dysfunction. A previous study observed the apoptosis of human MSCs induced by ROS after exposure to para-cresol, a uremic toxin, which was associated with reduced levels of PrP^C^ [8]. Although there were differences in other clinical attributes, such as age and body mass index, our previous studies confirmed that serum from CKD patients displayed a lower level of PrP^C^ compared with serum from healthy controls [31,32,33,34]. These results are consistent with the present observations of the association of decreased expression of PrP^C^ in CKD-MSCs with enhanced cellular ROS production, which resulted in the attenuation of stem cell function by activation of ER stress and dysfunctional mitochondria. ER directly communicates with the mitochondrial surface through a mitochondrial-associated membrane [48,49]. This cross-talk is mediated by mitochondrial-shaping proteins and key chaperones, such as calnexin, calreticulin, ER resident protein 44 (ERp44), ERp57, mitochondrial 70 kDa heat shock protein (mtHsp70), and the sigma-1 receptor [50]; these responses are also associated with ion channel and transporter proteins, ubiquitin ligases, vesicular-sorting proteins, electron transport chain proteins, and mitochondrial fusion proteins [50,51]. In particular, activation of PERK, IRE1α, and ATF6 induces altered communication between ER and mitochondria, leading to mitochondrial dysfunction, metabolic imbalance, and ultimately cell death. We observed enhanced activation of PERK, eIF2α, ATF4, and IRE1α, and mitochondrial fusion proteins like DRP1, MFN1, and OPA1 in CKD-MSCs. Pioglitazone inhibited the activation of ER stress sensor proteins and mitochondrial fusion proteins through the upregulation of PrP^C^. These findings implicate PrP^C^ as a therapeutic target for the protection of mitochondria against CKD-induced ER stress.

**Figure 6:**
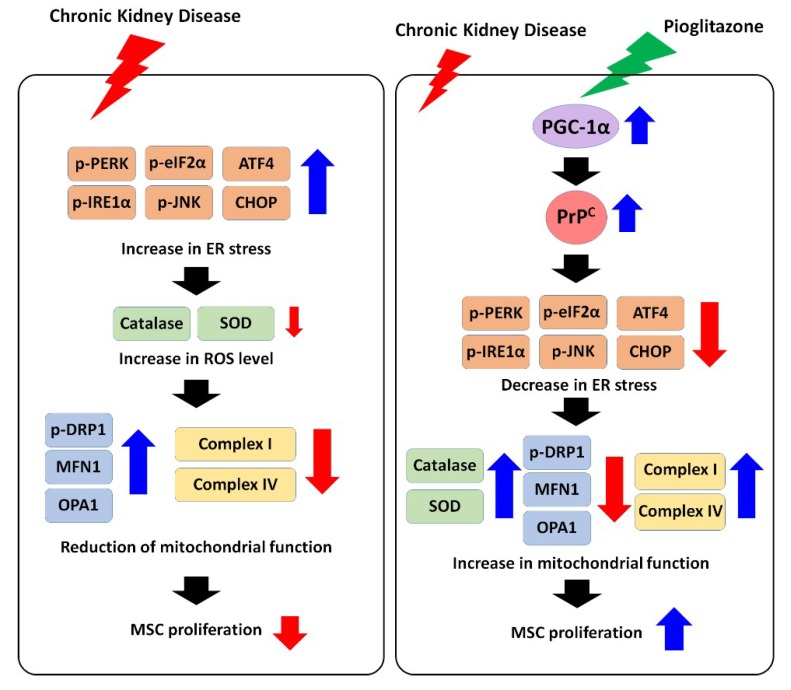
Scheme of the effect of pioglitazone on CKD-mediated ER stress in CKD-MSCs. CKD pathophysiological condition induces ER stress, ROS generation, and mitochondrial dysfunction, leading to reduction of cell proliferation in CKD-MSCs. However, treatment of CKD-MSCs with pioglitazone increases the activation of the PGC-1α-PrP^C^ signal pathway, resulting in protection of mitochondrial function and cell proliferation against CKD-induced ER stress. Black arrow means cell signal pathway. Red arrow means down-regulation of protein activation or expression. Blue arrow means up-regulation of protein activation or expression.

Treatment of CKD-MSCs with pioglitazone enhanced mitochondrial biogenesis and activity of complexes I and IV, which were comparable to those observed in healthy-MSCs. However, blocking PrP^C^ expression in CKD-MSCs inhibited the beneficial effects of pioglitazone. In particular, mitochondrial energy metabolism was affected, which is associated with adenosine triphosphate production and cell proliferation (Figure 6). The data demonstrate that pioglitazone protects CKD-MSCs from the inhibition of cell proliferation through PrP^C^-cyclin-dependent kinase 4 (CDK4) activation. Moreover, other studies have revealed that PrP^C^ is a key molecule for the proliferation of MSCs under several pathophysiological conditions, such as ischemic diseases, hypoxia, and uremic toxin exposure [13,27,52]. These findings suggest that PrP^C^ is a pivotal signaling molecule for MSC proliferation in CKD.

## 4. Materials and Methods

### 4.1. Human MSC Cultures

The study was approved by the local ethics committee of Soonchunhyang University Seoul Hospital (IRB: SCHUH 2015-11-017, Nov. 2 2015). Adipose tissue derived human MSCs (healthy-hMSCs CKD-hMSCs) were obtained from allogeneic donors (Table 1) following informed consent. CKD patients had an estimated glomerular filtration rate (eGFR) of < 35 mL/min/1.73 m^2^ (stage 3b) for more than three months. Briefly, MSCs isolated from adipose tissue of healthy volunteers and CKD patients were cultured in α-Minimum Essential Medium (α-MEM; Gibco BRL, Gaithersburg, MD, USA) supplemented with 10% (*v*/*v*) fetal bovine serum (Gibco BRL) and 100 U/mL penicillin/streptomycin (Gibco BRL) at 37 °C in an atmosphere of 5% CO_2_ overnight. Healthy-MSCs and CKD-MSCs from passage 3 to 5 were used. Human MSCs were identified by the expression of positive cell surface markers CD44 and Sca-1 and expression of negative cell surface markers CD45 and CD11b, and were differentiated into chondrogenic, adipogenic, and osteogenic cells under a specific differentiation media condition that we have previously described [31].

### 4.2. Western Blotting

Proteins (30 μg) in the cell lysate of healthy-MSCs and CKD-MSCs were resolved by 8–12% sodium dodecyl sulfate-polyacrylamide gel electrophoresis. The resolved proteins were transferred to an 8 μm pore size nitrocellulose membrane. The membrane was blocked using 5% skim milk prepared in TBST (10 mM Tris-HCl (pH 7.6), 150 mM NaCl, 0.05% (*v*/*v*) Tween 20) for 1 h at room temperature. The membrane was then treated with primary antibodies. The antibodies used were directed against p-PERK, anti-PERK, p-JNK, JNK, MFN1, PrP^C^, and β-actin (all 1:300 dilution, all from Santa Cruz Biotechnology, Dallas, TX, USA), p-eIF2α, eIF2α, ATF4, p-DRP1 (both 1:1000 dilution, both from Abcam, Cambridge, UK), p-IRE1α, IRE1α, CHOP, OPA1, and PGC-1α (all 1:1000 dilution, all from Novus, Centennial, CO, USA). Next, each membrane was washed twice, and the primary antibodies were detected using goat anti-rabbit IgG or goat anti-mouse IgG antibodies (Santa Cruz Biotechnology). The protein bands were detected by enhanced chemiluminescence (Amersham Pharmacia Biotech, Little Chalfont, UK).

### 4.3. Measurement of Mitochondrial Superoxide (O_2_^•−^) Production

O_2_^•−^ of healthy-MSCs or CKD-MSCs was measured using MitoSOX (Thermo Fisher Scientific, Waltham, MA, USA). The cells in each group were subjected to trypsinization and centrifuged at 600 g for 5 min. The samples were washed and incubated with 10 μM MitoSOX solution in phosphate buffered saline (PBS) at 37 °C for 15 min. Next, the cells were resuspended in 500 μL PBS, and the total number of cells labeled by MitoSOX was measured by fluorescence-activated cell sorting (Sysmex, Kobe, Japan). MitoSOX-positive cells were identified using Flowing Software (DeNovo Software, Los Angeles, CA, USA).

### 4.4. Immunofluorescence Staining

Cells were plated on a cover glass and fixed with 4% paraformaldehyde (Sigma-Aldrich, St. Louis, MO, USA). Immunofluorescence staining was performed using MitoTracker (Thermo Fisher Scientific). Nuclei were stained with 4′,6-diamidino-2-phenylindole (DAPI; Sigma-Aldrich). The stained samples were examined by confocal microscopy (Olympus, Tokyo, Japan).

### 4.5. Superoxide Dismutase (SOD) Activity

Protein from healthy-MSCs or CKD-MSCs was extracted using a RIPA extraction buffer (Thermo Fisher Scientific). SOD activity was measured using a SOD activity kit (Enzo, Basel, Switzerland). Briefly, 40 µg protein was added to each well. A master mix (150 µL) containing WST-1 reagent and xanthine oxidase was also added to each well. In this colorimetric assay, superoxide ions are generated from the xanthine oxidase catalyzed conversion of xanthine and oxygen to uric acid and hydrogen peroxide (H_2_O_2_). The superoxide anion then converts WST-1 to the color WST-1 formazan product. Following the addition of the xanthine oxidase solution (25 µL/well), the absorbance was measured at 450 nm every minute for 15 min using a microplate reader (BMG Labtech, Ortenberg, Germany). SOD activity was calculated following the manufacturer’s instructions.

### 4.6. Catalase Activity

Proteins isolated from healthy-MSCs and CKD-MSCs (40 µg) were incubated with 20 mM H_2_O_2_ for 30 min. This was followed by the addition of 50 mM Amplex Red reagent and 0.2 U/mL of horseradish peroxidase (Sigma-Aldrich), and incubation for 15 min at room temperature. Changes in the absorbance value associated with H_2_O_2_ degradation were measured using an Enzyme-linked immune sorbent assay (ELISA) plate reader (BMG Labtech) at 563 nm.

### 4.7. Electron Transport Chain Complex I Activity Assay

Complex I activity was measured using a complex I enzyme activity assay kit (Abcam) following the manufacturer’s instructions. Briefly, cell extraction proteins (125–1250 µg/mL) were added to each well of a microplate followed by incubation for 3 h at room temperature. Each well was washed three times. The mixture was diluted with dilution buffer to yield 20× nicotinamide adenine dinucleotide hydrogen (NADH) and 100× dye. The mixture was carefully added to each well (200 µL per well). The absorbance was immediately measured at 450 nm every minute for 30 min using an ELISA plate reader (BMG Labtech). Raw data were expressed as the rate (mOD/min) per µg/mL of cell lysate.

### 4.8. Electron Transport Chain Complex IV Activity Assay

Complex IV activity was measured using a complex IV enzyme activity assay kit (Abcam) following the manufacturer’s instructions. Briefly, each sample (5 mg/mL) was added to wells of a microplate and incubated for 3 h at room temperature. The bound monoclonal antibody immobilized the enzyme in the wells. Each well was washed three times using potassium phosphate buffer. The solution was removed and replaced with 200 µL of the assay solution containing potassium phosphate buffer and cytochrome complex (cyt c). The absorbance was measured every 1 to 5 min for 2 h at 550 nm using the ELISA plate reader (BMG Labtech). Complex IV activity was calculated as (absorbance at time 1 − absorbance at time 2)/Δ*t* (min). Since the initial rate was decreased due to the inhibited complex IV reaction, the rate of activity was always expressed as the initial rate of oxidation of cyt c.

### 4.9. Cyclin-Dependent Kinase 4 (CDK 4) Kinase Assay

A cyclin-dependent kinase 4 (CDK 4) kinase assay was performed using a CDK 4 Kinase Assay Kit following the manufacturer’s instruction (Cusabio, Baltimore, MD, USA). Briefly, the standard and sample were added to each well of a microplate (100 µL) for 2 h at 37 °C. After aspiration of the fluid from each well, 100 µL of biotin-conjugated antibody was added for 1 h at 37 °C. Each well was then washed twice using washing buffer followed by the addition of 100 µL of horseradish peroxidase-avidin to each well for 1 h at 37 °C. Each well was then washed five times, followed by the addition of 90 µL of 3,3′,5,5′-tetramethylbenzidine substrate to each well for 15 to 30 min in the dark. Stop solution (50 µL) was added to each well and the absorbance was determined at 450 nm using the ELISA plate reader (BMG Labtech).

### 4.10. Cell Proliferation Assay

Healthy-MSCs or CKD-MSCs were cultured in 96-well culture plates (3000 cells/well) and treated with 5-bromo-2′-deoxyuridine (BrdU). The BrdU incorporated into the newly synthesized DNA of the proliferating cells. These cells were assessed using a BrdU ELISA colorimetric kit (Roche, Basel, Switzerland). Briefly, 100 μg/mL BrdU was added to each well and incubated at 37 °C for 3 h. The medium containing the BrdU labeling solution was removed and FixDenat (200 μL) was added. The microplate was incubated at room temperature for 30 min. Intact DNA in the wells containing healthy-MSCs or CKD-MSCs was labeled by BrdU during this period. Anti-BrdU antibody (100 μL) was added to each of these wells and incubated at room temperature for 90 min. The cells were then treated with 100 μL of 3,3′,5,5′-tetramethylbenzidine solution at room temperature for 20 min. Absorbance was measured using the microplate reader (BMG Labtech) at 370 nm.

### 4.11. Cell Cycle Analysis

Healthy-MSCs or CKD-MSCs were harvested and fixed with 70% ethanol at −20 °C for at least 2 h. The cells were washed twice with cold PBS and incubated with RNase and a DNA-intercalating dye, propidium iodide (Sysmex, Kobe, Japan), at room temperature for 30 min. The cell cycle of the propidium iodide-stained cells was characterized by fluorescence-activated cell sorting (Sysmex). Events were recorded for at least 10^4^ cells per sample and the experiment was repeated three times. The data were analyzed using FCS Express 5 software (DeNovo Software).

### 4.12. Statistical Analyses

Results have been expressed as mean ± standard error of the mean (SEM). The significance between groups was tested by a two-tailed student’s *t* test or by one- or two- way analysis of variance (ANOVA). Comparison between three or more groups was made using a Dunnett’s or Tukey’s post-hoc test. Data were considered significantly different at *p* < 0.05.

## Figures and Tables

**Table 1:** Demographic and clinical characteristics and estimated glomerular filtration rate (eGFR) of patients.

Healthy-MSCs			CKD-MSCs		
Gender	Age, Years	eGFR (mL/min/1.73 m^2^)	Gender	Age, Years	eGFR (mL/min/1.73 m^2^)
F	62	125.03	F	37	43
F	39	101.62	M	68	28
M	63	108.84	F	43	21
M	54	91.17	F	51	11
F	39	86.44	M	39	9
